# Examining the impact of text style and epistemic beliefs on conceptual change

**DOI:** 10.1371/journal.pone.0220766

**Published:** 2019-09-04

**Authors:** Angele Yazbec, Arielle Borovsky, Michael P. Kaschak

**Affiliations:** 1 Department of Psychology, Florida State University, Tallahassee, FL, United States of America; 2 Department of Speech, Language, and Hearing Science, Purdue University, West Lafayette, IN, United States of America; Jacobs University Bremen, GERMANY

## Abstract

Learning can be difficult for students due to incorrect prior knowledge, or misconceptions, interfering with the acquisition of new knowledge. Conceptual change refers to the process of replacing such misconceptions with new and accurate knowledge. The factors associated with conceptual change are currently under debate. The present study attempts to replicate previous investigations of how text style and epistemic beliefs impact conceptual change, and extends this work by investigating how those factors differentially facilitate conceptual change within participants. 157 college students completed a two-part, within participants study in which they completed pretests, read passages addressing a misconception, completed posttests, and were assessed on their epistemic beliefs. Text style (expository vs. refutation) and two dimensions of epistemic beliefs (texture and variability) did not directly impact pre-to-posttest changes in performance. However, interactions between text type, texture, and variability were related to changes in performance.

## Introduction

One reason students struggle with learning is they have misconceptions about how the world operates (e.g. [1,2,3,4,5]). For example, many people have misconceptions about motion and other physical dynamics stemming from observational attributions of causality in the environment [6]. These inferences and naïve conceptualizations about how the world operates, however, may be inconsistent with current scientific understanding of natural phenomena [7]. These misconceptions are problematic because they contradict scientific evidence on the subject, and can be resistant to change since they are strongly held beliefs about the world (e.g. [3,8]). Although prior knowledge is often useful in comprehending text (e.g. [9,10]), misconceptions contain inaccurate information and create conflict with text encountered in educational settings. The process of outdating these misconceptions with new, more accurate information is referred to as conceptual change (e.g. [11,12,13]).

Many studies highlight the factors that influence conceptual change, but to what extent they influence this process is not entirely clear. The divergence of findings in conceptual change can be partially attributed to the wide range of academic disciplines that have examined conceptual change, but take different approaches on how to facilitate the process (e.g. [14]). The present study investigates the elements of text that encourage conceptual change, and how readers’ epistemic beliefs (beliefs about the nature of knowledge and the process of knowing; [15]) can influence whether and to what extent conceptual change occurs when reading science text. The present investigation also involves an exploration of additional individual differences that may facilitate the process of conceptual change.

### History and overview of conceptual change

While the investigation of conceptual change is not new to the field, there is not yet a unified framework for approaching the study of conceptual change. Many early misconception studies were even devoid of unified theory (for review see [14; 16]). Presently, there are two broad theoretical approaches on knowledge structure that are applied to this area of study: (1) knowledge-as-theory and (2) knowledge-as-pieces [16]. The knowledge-as-theory perspectives are widely influenced by Piaget’s assimilation and accommodation concepts. One of the most widely noted models of conceptual change within the knowledge-as theory-perspective is the Conceptual Change Model (CCM) proposed by Posner and colleagues (1982). According to this model, in order for conceptual change to occur, the following must occur: (1) the learner becomes dissatisfied with their prior knowledge, (2) the new concept is intelligible and plausible to the learner, and (3) the new concept must be fruitful for explanation [13]. Much of the prior research is based on the idea that learners hold an incorrect theory that must be corrected in light of new information (e.g. [1,5,17]). In contrast to the knowledge-as-theory approach is the knowledge-in-pieces approach, in which learners’ understanding is based on a collection of multiple, semi-independent elements of knowledge (e.g. [16,18,19]). According to the knowledge-in-pieces perspective, learners may hold intuitive ideas that do not need to be rejected, but rather can be incorporated into a stronger conceptual factor that is reinforced through learning [18,19].

The knowledge-as-theory and knowledge-in-pieces perspectives both offer insights into the process of conceptual change. However, it is not our intent to put these approaches to the test in the present study. Rather, our goal is to provide an empirical investigation of two factors that have been implicated in conceptual change. The first factor is text structure, particularly the question of whether expository or refutation texts are better for outdating misconceptions. The second factor is the learner’s epistemic beliefs (e.g., beliefs about knowledge, and how changeable it is). These factors have been explored in previous research [20,21,22], but as we discuss below, some questions remain about the impact that each factor has on conceptual change.

Before moving on to a discussion of text structure and epistemic beliefs, we first provide our working definition of conceptual change. For our purposes, conceptual change refers to whether a learner, who previously held a misconception, will successfully acquire new knowledge that refutes the misconception after reading text in support of new knowledge. This view of conceptual change can be operationalized as the difference in performance between a pre- and post-test in the relevant knowledge domain.

### Designing optimal text

Successful text comprehension involves constructing a mental representation of the text and continuously updating it as the text unfolds. Mental representations of the text are augmented by prior knowledge, resulting in a model that blends the learner’s existing knowledge with input from the current text [9,10]. One approach to facilitating conceptual change in science is to ensure the text highlights the new concept in a way that the learner recognizes that the misconception is inadequate for explaining scientific phenomena, and that the new ideas are useful for explaining phenomena [13]. It has been argued under the Knowledge Revision Components (KReC) Framework that a key step to encouraging conceptual change (also referred to as knowledge revision in this literature) is making the learner explicitly aware there is a conflict between previously acquired knowledge and new information [23]. The awareness component is critical because the old and new ideas must be co-activated to induce cognitive conflict. Learners must determine what happens in the presence of conflict. In successful cases of conceptual change, learners will integrate the new information into their knowledge structure, thus decreasing activation of the misconception [23].

There evidence suggesting the quality and quantity of explanation matters in order for conceptual change to occur. Initial investigation of the text factors that promote knowledge was in the domain of narrative text comprehension (e.g. [24,25,26,27]). In the inconsistency paradigm, a character is introduced as having a certain behavior (e.g. *“Mary*, *a health nut*, *has been a strict vegetarian for ten years*.*”*) ([27], p. 1209). At the end of the narrative, however, a critical sentence reveals the character engaging in a behavior that is inconsistent with the behavior described in the beginning of the text (e.g. *“Mary ordered a cheeseburger and fries*.*”*) ([27], p. 1209). In between the introduction and the last sentence were the key manipulations of different explanations of the character’s behavior that would possibly make that critical sentence less disruptive to the reader. Findings suggest that causal explanations of character’s behavior lead to less disruption in comprehension compared to qualified explanations, as indexed by lower reading times in the causal explanation condition [25]. The quantity of explanation also makes a difference in how much conceptual change the reader exhibits. Explanations that provide an explanation for why a behavior changed (e.g. *“She wasn’t getting enough vitamins because of her diet so her doctor said she had to start eating meat*.*”*) ([25], p. 865) showed less disruption of an old characteristic compared to an explanation that simply outdates the old behavior (e.g. “*Recently Mary had stopped worrying about eating nutritious foods and began to eat more meat and fast food*.*”*) ([27], p. 1209). Thus, causal explanations could help learners undergo more conceptual change than qualified explanations because there is more information substantiating why the new idea holds more support than the old one.

There are many different styles of text, but the main ones of interest in the conceptual change literature are refutation and expository text. Expository text is the text style that is typically encountered in textbooks: it disseminates factual knowledge. Refutation text structure is similar to expository text with one notable difference: it explicitly highlights a misconception. Consider an example in which the targeted misconception is “meteors that land on Earth are hot.” Both refutation and expository texts explain that the outer molten layer of a meteor burns off while entering Earth’s atmosphere, and the meteor does not have time to heat up before it lands. The key difference between these texts will be in how this explanation is prefaced. Refutation texts may start with a preface such as, “*Kate warned everyone not to touch the meteor because it would be hot and they could get burned*. *However*, *Jerry said that they should not worry because it actually should not be hot*.” ([28], p. 396). This preface explicitly states that meteors are hot when they land on Earth is a misconception. Expository text, on the other hand, does not explicitly point out the misconception. For example, the preface may be something like this: “*Kate was excited and curious because she had never seen a meteor on the ground before*. *Jerry said he could look up more about meteors in the astrophysics book that he had*.” ([28], p. 396).

A number of studies have found that refutation text is better at inducing conceptual change than expository text among school-aged and college students (see [29] for review, [30]). However, a smaller number of studies find the two styles do not differ in inducing conceptual change (e.g. [31,32]). Divergence in these findings can be attributed to differences in structuring the two text styles and different methodologies, such as reading times (e.g. [28,33]), protocol analysis or think alouds (e.g. [34,35,36]), and eye tracking (e.g. [32,37]). Despite the conflicting evidence, the prevailing view in the field remains that refutation texts are better for conceptual change than expository text for students from elementary school through university (e.g. [19,38]). The main argument is when refutation text has its optimal characteristics of including an explicit statement that a misconception is false, an explicit statement that the new idea is true, and there is sufficient supporting evidence favoring the new idea (e.g. a substantive causal explanation), a reader will exhibit more conceptual change than when reading an expository text (e.g. [23,28]). While refutation text could help facilitate conceptual change, additional reader characteristics, such as epistemic beliefs could illuminate key factors that are important for conceptual change. We review this topic in the next section.

### Role of epistemic beliefs

Even with the best-designed text structure, conceptual change may also depend on the learner’s ability to engage in conceptual change. Epistemic beliefs–individual beliefs about the nature of knowledge and the process of knowing—are one of the more interesting characteristics (e.g. [21,39,40,41]). There are many different ways to approach and classify epistemic beliefs. For instance, there are the terms “naïve—sophisticated” (e.g. [42]) and “less advanced—more advanced” (e.g. [32]). Naïve and sophisticated refer to the extent to which a learner believes knowledge is stable versus subject to change. For example, a learner with more sophisticated epistemic beliefs often perceive knowledge as tentative and subject to change whereas a learner with naïve epistemic beliefs tend to perceive knowledge as stable and unchanging [42].

It has been noted that students with more naïve epistemic beliefs have faced more challenges to learning than students with complex epistemic beliefs (see [22] for review). Previous studies have noted that students with more advanced epistemic beliefs exhibited more conceptual change at the immediate posttest compared to students who had less advanced epistemic beliefs, which is consistent with other findings on this relation [20,43,44,45]. An investigation of the interaction of text with epistemic beliefs showed that among students reading refutation text, those with more advanced epistemic beliefs demonstrated more conceptual change than students with less advanced epistemic beliefs [20]. This interaction, however, was not observed with expository text: there were no differences in conceptual change between students with more advanced epistemic beliefs compared to those with less advanced epistemic beliefs. These patterns persisted into the delayed posttest, even though the scores were overall lower than the immediate posttest [20].

Despite this common finding in the literature on conceptual change and epistemic beliefs, it is important to note that it may not always be the case that having more sophisticated epistemic beliefs will lead to an advantage in learning, or even conceptual change, compared to having naïve epistemic beliefs. The view of naïve—sophisticated, or certain—uncertain, epistemic beliefs has been argued to be insufficient as the context for learning itself could modify when it would be more appropriate to have naïve (or certain) or sophisticated (or tentative) epistemic beliefs. Elby and Hammer (2001) point out that it is not correct to broadly apply the principle *knowledge is tentative* to every concept, as the context for learning may call for an approach that knowledge is certain. For example, when elementary school students learn the Earth is flat, it is neither correct nor productive for them to approach knowledge as tentative since the Earth being round is a verifiable fact. Thus, it is more productive for students to accept that, in this instance, knowledge is certain and learn the fact the Earth is round. For theories of dinosaur extinction, on the other hand, it would be more productive for students to take a tentative approach to knowledge as that information is not definitive and subject to revision, especially when new scientific evidence can present evidence that conflicts with previous knowledge in the field [42].

Some research suggests that aligning text to learner’s epistemic beliefs may be more beneficial to conceptual change than just having sophisticated epistemic beliefs. Franco and colleagues (2012) found that learners with a rational approach to epistemic beliefs (e.g. belief that knowledge is derived with reason and logic) exhibited more conceptual change when given a text with a rational approach compared to a metaphorical approach (e.g. belief that knowledge is derived via intuition). Similarly, learners with a metaphorical approach to epistemic beliefs exhibited more conceptual change when reading text with a metaphorical approach compared to a rational approach [21]. People’s current topic domain knowledge may also play a role in their epistemology. For instance, someone with little knowledge about physics in general may perceive the field as precise due to the methods used to measure physical quantities. On the other hand, someone with extensive knowledge about research in physics may perceive the field as imprecise because various methods are still in development [46]. Furthermore, there are also ontological differences in epistemology depending on the discipline examined. For example, knowledge in math and science is often considered to be more certain than knowledge in psychology or history because of the different ways with which knowledge is dealt across disciplines [46]. Thus, optimal learning in different disciplines may require less advanced epistemic beliefs compared to others. For example, a learner understanding mathematical processes would benefit more from less advanced epistemic beliefs because it would be more productive to learn the process rather than be open to alternatives that could lead to inaccurate answers. On the other hand for history, new archaeological and anthropological evidence may present new information about a historical time period that conflicts with previous knowledge about that era; therefore, it would be more fruitful for the learner to understand that knowledge in history is uncertain and be more adaptable to learning the new information.

While there are different approaches to defining epistemic beliefs, in the present work, we will refer to epistemic beliefs on the spectrum of less advanced to more advanced (see [20] for similar approach to classifying epistemic beliefs). As epistemic beliefs were measured using the Connotative Aspects of Epistemic Beliefs questionnaire [47], we were tapping into two different aspects of epistemic beliefs: texture and variability. Texture refers to the extent to which learners believe knowledge to be unstructured and vague. Variability refers to the extent to which students believe knowledge to be flexible and dynamic. Following the guidelines from previous studies (e.g. [20,47]), in this study, more advance epistemic beliefs will refer to higher scores in texture while less advance epistemic beliefs will refer to higher scores in variability. Our understanding of the relationship between individual characteristics of the learner and the nature of the learning materials (such as the text that is being read) is still emerging and requires further investigation [20,21,22,43,44,45]. Thus, while epistemic beliefs appear to play a role in conceptual change, its role is still not well understood. Furthermore, it is important to understand the extent to which having more or less sophisticated/advanced epistemic beliefs helps or hinders the learner from undergoing conceptual change in different disciplines.

### Present study

The existing literature points to the importance of text type and epistemic beliefs in promoting conceptual change. However, there is conflicting evidence as to whether or not refutation text is better at inducing conceptual change in readers compared to expository text. Much of this evidence comes from studies with between subjects designs (e.g. [20,37,40,48]), which limits insight as to how individual readers may approach the different styles of text, and subsequently, how much they learn from them. This study aims to replicate previous studies investigating how text style and epistemic beliefs influence conceptual change (e.g. [20]), and also extend those findings by conducting a within subjects investigation to determine if an individual reader exhibits different amounts of conceptual change after reading a refutation text versus an expository text.

The primary aim of this study is to investigate whether and how the interaction of text style (refutation versus expository) and epistemic beliefs (ranging from less advanced to more advanced, per the guidelines suggested in Mason and Gava, 2007 [20] for describing the continuum of epistemic beliefs) impacts conceptual change in different subject areas. If text style and epistemic beliefs play a role in conceptual change, then it is expected that refutation text will induce more conceptual change than expository text. Additionally, learners who score higher in texture and variability will exhibit more conceptual change than learners who score lower in texture and variability.

## Materials and methods

### Preregistration disclosure

The study was preregistered on Open Science Framework (OSF): osf.io/msq7w. The methods and model described below are in accordance with the preregistration. Any divergence or elaboration from the preregistration on OSF is noted. The data from this study is available on the OSF website.

### Participants

The Florida State University Office of the Vice President for Research Human Subjects Committee approved this study on 6/8/2017 (Approval number: 2017.21233) and renewed it on 3/21/2018 (Approval number: 2018.23500). Consent was obtained through a written form requiring the participant's signature before starting the study. One hundred and seventy-two college-aged participants were recruited from Florida State University’s Department of Psychology and were compensated with course credit for participation (*M =* 19.28 years, male = 37). 157 participants completed both sessions and were included in the final sample. This sample size was generated following the sequential sampling guidelines described in Frick (1998), which allow researchers to be flexible and efficient in their sample sizes while also controlling for Type I errors [49]. We proposed an initial target sample size of 100 college-aged adults. Once this sample sized was reached, we did preliminary data analyses to determine whether the critical effects (involving text type and epistemic beliefs) had *p*-values greater than .35 or less than .01 (e.g., Frick’s stopping rule). As the effects did not meet this criterion, we continued data collection toward our pre-registered maximum sample size of 150. We collected data from more than 150 participants in an effort to ensure that our final sample had more than 150 participants once participant attrition was taken into account.

### Text

Following a similar procedure outlined by Lassonde and colleagues [50], refutation and expository texts were written for each topic (see S1 File for example). Five disciplines were included (physics, astronomy, genetics, economics, and geography) and each discipline had two topics that were tested (see Table 1).

**Table 1:** List of disciplines and topics assessed in the study.

Discipline	Topic
Physics	Newton’s 1^st^ law of motion
	Newton’s 3^rd^ law of motion
Astronomy	Star formation
	Star colors
Genetics	Alleles
	Chromosome
Economics	Inflation
	Gambler’s fallacy
Geography	World geography
	Floridian geography

All texts contained the following sections: introduction, premise, explanation, and conclusion. The key difference between the refutation and expository versions of the texts for each topic was the premise section. The premise section for the refutation text explicitly stated the misconception and noted the misconception is incorrect (see example below).

Many people think red stars are hotter than blue stars because red is often associated with fire and other notably hot surfaces or objects. Astronomers, however, have found this idea to be inaccurate. Quantitative measures of starlight color and stellar temperature reveal that blue stars are actually the hottest stars while red stars are the coolest stars.

The premise section for the expository text presented further information about the topic, but does not explicitly state the misconception (see example below).

Whether or not blue stars or red stars are the hottest is not that clear to the casual stargazer. Close investigation of the visible light spectrum of stars, however, does reveal vast temperature differences. Astronomers have devised measures for quantifying the colors of light the stars give off and then using those colors to determine stellar temperatures.

We carefully controlled the length of each text and its subsections. Across all texts, there was a range of 346–349 words. The premise section for each version of each text was within one word of each other. The introduction, explanation, and conclusion sections were exactly the same length for each text version. We also ran the Flesch Reading Ease and Flesch-Kincaid Grade Level tests to obtain information about the reading level of each text (see Table 2 for results). Higher Flesch Reading Ease scores indicate the text is easier to read [51], while higher Flesch-Kincaid Grade scores indicate the text is appropriate for the given numerical grade level in the U.S. [52]. It is generally recommended that most standard text in the U.S. should score between 60–70. Overall, the text ranged from 10^th^ grade to first year of college reading level and the texts were generally more difficult read.

**Table 2:** Results from the Flesch Reading Ease and Flesch-Kincaid Grade Level tests.

Discipline and Topic				
	Refutation		Expository	
	Flesch Reading Ease	Flesch-Kincaid Grade Level	Flesch Reading Ease	Flesch-Kincaid Grade Level
Physics				
Newton’s First Law of Motion	46.4	11.8	46.2	12.1
Newton’s Third Law of Motion	50.7	11.9	48.2	12.2
Astronomy				
Star Colors	48.7	11.2	49.0	11.2
Star Formation	42.9	12.3	42.4	12.4
Genetics				
Alleles	42.3	12.1	38.1	12.7
Chromosomes	49.6	10.2	50.0	10.5
Economics				
Inflation	45.4	12.0	47.0	12.1
Gambler’s Fallacy	43.7	11.7	46.3	11.3
Geography				
Florida Geography	29.4	13.9	30.4	14.0
World Geography	28.4	13.8	30.3	13.8

### Experiment measures

#### Pretests and posttests

Pretests and posttests were the same for each topic. Test questions for the physics topics were pulled from the Force Concept Inventory [53] and test questions for the astronomy topics were pulled from the Star Properties Concept Inventory [54]. Items for the genetics questions were taken from the Genetics concepts inventory [55]. Items for economics were taken items assessing knowledge of inflation from the Economic model questionnaire [56] and the gambler’s fallacy test [57]. All items pulled from the Force Concept Inventory, Star Properties Concept Inventory, Genetics concepts inventory, Economic model questionnaire, and the gambler’s fallacy test multiple-choice questions with one correct answer.

The global geography questions were pulled directly from the compass direction task listed in Tversky [58] and the Florida geography questions were constructed by the principal investigator in a similar style (S1 Fig). Because we were administering the tasks electronically, we adapted the compass direction task to Qualtrics. Participants were given a set of two cities (e.g. Philadelphia-Rome). Then they were asked to imagine the center dot was the first city (e.g. Philadelphia) and then were asked to click where in the circle the second city (e.g. Rome) was located in relation to the first city. There was one region coded as correct and all responses falling outside of that area were coded as correct. For example, the correct answer for where Rome is located in relation to Philadelphia is that Rome is northeast of Philadelphia. Responses within the southwest portion (e.g. lower left-hand part) of the circle were coded as correct whereas responses outside of that region were incorrect.

The topics were split such that each discipline would have one topic tested at the first session and one topic tested at the second session. The number of questions in the pretests and posttests for across topics sets one and two were close to equal (30 or 33 questions, respectively).

#### Epistemic beliefs

Epistemic beliefs were measured using the Connotative Aspects of Epistemic Beliefs (CAEB) scale developed by Stahl and Bromme (2007) [5]). This inventory starts with an opening statement (e.g. “Knowledge in physics is…”) and includes 24 adjective pairs (e.g. exact—vague) that could be used to describe knowledge in that given discipline on a scale of 1–7. For this scale, “1” (e.g. exact) aligns with a less advanced epistemic belief and “7” (e.g. vague) aligns with a more advanced epistemic belief. In accordance to the original scale, some items were reverse coded. Participants filled out one CAEB scale per discipline (physics, astronomy, genetics, economics, and geography, respectively). The items on the CAEB were then split into texture and variability as these are the two dimensions of epistemic beliefs captured by this measure (see [41] for further explanation). Following the results of the two-factor analysis outlined by Stahl and Bromme (2007) and Pieschl and colleagues (2008), a summed score for texture and a summed score for variability were computed. There were individual scores for texture and variability computed for each discipline, and then a single texture and a single variability score were computed summing the texture and variability scores from each discipline.

### Procedure

This study was a two-part, within-subjects design. Participants were informed in advance they would be participating in a two-session study with each session taking place approximately one week apart, lasting about one hour each. Time lag between sessions ranged from 5–9 days. During the first session, participants completed the CAEB scales, completed a pretest on one of the topics from each of the five disciplines, read text concerning the topics, and completed an immediate posttest. All tasks were completed on a Qualtrics survey that was saved on lab computers and links were not externally shared or available to anyone aside from the principal investigator. To avoid carryover effects of text style, participants read either (1) expository or (2) refutation text during the first session.

The second session was completed in a separate room in the lab, and participants read text on different topics from the same five disciplines, but in style they did not read during the first session. The second session had a similar structure with participants completing a pretest on different topics from the first session, reading text, and completing an immediate posttest on a Qualtrics survey. Participants would complete a pretest, text reading, and posttest per discipline, one at a time. It is important to note in this within-subjects design, participants read text in a style that was different from style they read in the first session (e.g. if they read expository previously in session 1, they would read refutation text in session 2). The order of topics tested was randomized across participants and order of text style was counterbalanced across participants.

### Data coding

We coded our responses as correct (1) or incorrect (0). While we declared in our preregistration guidelines that missing items would be scored as incorrect, further examination of the data suggested that missing responses were largely a result of participants not finishing the task rather than skipping items themselves. In light of that finding, we report the models with missing responses removed. Items with missing responses were removed from analysis (100 items; 4.73% data loss).

The dependent measure for the study was response accuracy, which was dichotomously coded as either correct or incorrect at the item-level (1 = correct, 0 = incorrect). Each question included in this study was a multiple-choice question pulled from preexisting inventories, with the exception of geography which was developed by the primary author (see Experiment Materials, Pretests and Posttests for further information). Each came with an answer key (or one was developed by the primary author for the geography items). The answer keys noted which answers correct for each question. If the selected answer was not the correct one, it was coded as incorrect.

The following independent variables were also included in analysis: text type, test time, and epistemic beliefs. Text type was contrast coded for expository (-1) and refutation (1) text. Test time was also contrast coded for pretest (-1) and posttest (1). The two-factors for epistemic beliefs, texture and variability, were included as continuous variables. Both texture (α = 0.76) and variability (α = 0.38) were centered around their means prior to analysis.

### Data analysis

We ran descriptive statistics and reliabilities for the pretest and posttest for each topic areas as well as for texture and variability. Results can be seen in Table 2. The main statistical analysis comprised a mixed models logistic regression predicting the log odds of correctly answering a test question. Conceptual change would be demonstrated by a change in the log odds of answering a question correctly between the pre-test and post-test. The model included the following predictors (and interactions between these predictors): text type (refutation vs. expository), time (pretest vs. posttest), and texture and variability (continuous predictors). We expected that participants would generate more correct answers on the post-test (i.e., show more conceptual change) when they read refutation texts and score higher in texture and lower in variability than when they read expository text and scored lower in texture and higher in variability. The model also included participants and items as crossed random factors. The preregistration specified that we would run models with the full complement of random slopes and remove random slopes if necessary, to get the model to converge. Because we did not specify a method for removing random slopes, we decided use the model-fitting approach to compute a model with the best fitting random slopes.

## Results

We first tested the hypothesis that conceptual change will be greater after reading refutation texts as opposed to expository texts and learners who score higher in texture and variability will undergo more conceptual change than learners who score lower in texture and variability. For the main model we analyzed the complete data set, collapsing across disciplines (see Table 3). We looked at the accuracy for all items on the pretest, and there were no items at or near ceiling (*M =* 0.38, *SD =* 0.48), which indicates that participants had potential to learn from the text. We calculated Cronbach’s alpha to assess the reliability of the knowledge test as a whole, and within each individual subdomain. The results from this analysis were difficult to interpret, as a couple of features of our dataset made Cronbach’s alpha a less-than-ideal measure of reliability. First, our test did not have many items within each knowledge domain. Second, our items varied a good deal in terms of overall performance (some items were answered correctly far more often than others). Under these conditions, Cronbach’s alpha will not provide an accurate index of test reliability (see [59], along with discussion and references inside). For this reason, we do not report the alpha values for the pretests and posttests.

**Table 3:** Means and SD for texture and variability.

Discipline	Texture	Variability
All disciplines	154.27 (36.66)	141.18 (16.47)
Physics	28.45 (12.18)	26.59 (6.57)
Astronomy	35.96 (9.63)	32.47 (5.77)
Genetics	27.46 (8.74)	27.29 (5.41)
Economics	35.83 (9.96)	30.59 (6.20)
Geography	26.57 (9.05)	24.24 (7.61)

Results revealed a significant main effect of test time such that participants performed significantly better on the posttest compared to the pretest (Table 4; S2 Fig). The performance difference indicates that participants did in fact undergo conceptual change after reading text. Contrary to our hypothesis, neither text style, texture, nor variability alone were significant predictors of performance. There was, however, a significant interaction between text style and texture such that learners who read refutation texts and scored higher in texture exhibited more conceptual change than learners who read expository text and scored lower in texture. This finding aligns with our hypothesis, and suggest that refutation text, when combined with the approach that knowledge is complex, will lead to more conceptual change. Also in line with our hypothesis is a significant interaction between text style and variability such that learners who read refutation texts and scored higher in variability exhibited more conceptual change than learners who read expository text and scored lower in variability. While we find these patterns, it is important to keep in mind these interactions were rather small in magnitude; thus, they should be interpreted with caution. No other main effects or interactions were significant.

**Table 4a:** Results for the main model.

**Fixed Effects**				
Predictor	Coefficient	SE	t-value	p-value
Intercept	-0.420	0.136	-3.091	0.002
Texture	-0.001	0.001	-1.146	0.254
Variability	0.003	0.002	1.413	0.160
Text Style	0.012	0.034	0.360	0.719
**Test Time**	**0.217**	**0.053**	**4.134**	**< 0.001**
Text Style x Test Time	0.032	0.022	1.435	0.151
**Random Effects**				
Random Effect	Variance Comp.	St. Dev.	χ^2^-value	p-value
**Text Style x ID**	**0.080**	**0.283**	**521.551**	**< 0.001**
Test Time x ID	0.004	0.068	160.959	0.313
Text Style x Text Time x ID	<0.001	0.016	127.111	> 0.500
**Text Style x Item**	**0.025**	**0.157**	**139.216**	**< 0.001**
**Test Time x Item**	**0.157**	**0.396**	**609.779**	**< 0.001**
**Text Style x Test Time x Item**	**0.014**	**0.119**	**98.885**	**0.002**
**Text Style x Texture**	**<0.001**	**0.003**	**92.368**	**0.006**
**Text Style x Variability**	**<0.001**	**0.009**	**123.419**	**<0.001**
Test Time x Texture	<0.001	0.001	40.840	>0.500
Test Time x Variability	<0.001	0.003	40.678	>0.500
Text Style x Test Time x Texture	<0.001	0.002	57.662	>0.500
Text Style x Test Time x Variability	<0.001	0.003	52.774	> 0.500

^a^ID refers to participant.

We acknowledge that the individual disciplines included in the study could lend themselves to differences in difficulty in learning from the text, and that learners’ epistemic beliefs may vary across disciplines. Although we did not have any predictions about potential differences across academic disciplines, we conducted a set of exploratory analyses aimed at determining whether the patterns seen in the main analysis were also found within each discipline. The main results from these analyses are that (1) none of the disciplines showed differences in conceptual change between the refutational and expository texts, (2) the influence of variability on conceptual change was statistically significant such that learners who score higher in variability exhibited more conceptual change in some disciplines (astronomy, genetics, and economics), but not others (astronomy and physics), and (3) the influence of texture alone was not statistically significant across disciplines. A full report of these analyses is presented in the Supplemental Materials (Fig A in S2 File). We did some investigation of individual differences as well, but for the sake of brevity they are not reported in this paper. Results with individual differences can be found in our preregistration on OSF.

## Discussion

This investigation yields several important insights into how text style and epistemic beliefs support conceptual change. We tested two main hypotheses: (1) Refutation texts would promote greater conceptual change compared to expository text, and (2) Readers with more advanced epistemic beliefs would promote greater conceptual change compared to readers with less advanced epistemic beliefs. In general, participants increased their scores from pretest to posttest, which suggests that the experimental materials did promote conceptual change overall. However, conceptual change did not vary across text style alone (consistent with [30,31,32]). Rather our findings point to very divergent patterns across individual participants in learning from texts across disciplines. Several factors could explain these patterns, including the idea that different individuals require different types of explanations across domains, or that some patterns could be attributed to the text itself.

While we noted some interactions between text type and epistemic beliefs, we take caution in these findings given that the interactions are small in magnitude. In line with our hypothesis, the overall interaction shows that refutation texts and higher scores in texture and variability for epistemic beliefs are related to more conceptual change; however, this finding was not consistent across disciplines. Instances of successful conceptual change might be interpreted as supportive of the knowledge-as-theory approach to inducing conceptual change because change from incorrect answers on the pretest to correct answers on the posttest may indicate a successful replacement of previously incorrect knowledge; however, that does not necessarily mean we can rule out the knowledge-in-pieces perspective. While we noted conceptual change, it is possible that learners did not completely eschew their misconceptions, as would be the case with the knowledge-as-theory approach. Rather, it is possible readers incorporated the new information alongside with their misconception to create a stronger conceptual framework for the concept while not completely disregarding the misconception, which would be more consistent with the knowledge-as-pieces approach. On the other hand, for instances in which conceptual change was not observed (e.g. astronomy and physics in the present study), it is possible there was not enough information provided in the short reading passage to replace the incorrect knowledge, which would be in consistent the with knowledge-as-theory approach of needing to entirely replace the misconception with new and accurate information. However, we cannot necessarily rule out knowledge-as-pieces either as it could be the case that while misconceptions were noted, there was not enough time for a stronger conceptual framework containing both the misconception and the new information to be reinforced in time for learning on the posttest. Unfortunately, our methodology did not allow the opportunity for us to disentangle between these two perspectives for cases of successful and unsuccessful conceptual change. Prior studies have done some think alouds to measure what learners are thinking throughout the process of reading text and answering questions (e.g. [34,35,36]). Future work can incorporate think alouds to illuminate to what extent learners reject their misconceptions in favor of new knowledge (consistent with the knowledge-as-theory approach) versus creating a stronger conceptual framework containing both misconceptions and new information (consistent with the knowledge-as-pieces approach). Future work can also consider using open-ended responses to allow readers to demonstrate their understanding of the concept (e.g. [40]). This methodology could also help disentangle the extent to which misconceptions were debunked or attenuated but used alongside newly acquired knowledge after reading passages.

Previous work had suggested that not only the *type*, but also the *quality* of explanation matter for revising knowledge (e.g. [25,27]). As such, even when providing different explanations as to why a misconception is incorrect, it could be important to consider the quality of the explanation provided within the text, above and beyond just the style of text (e.g. refutation versus expository) by itself. Thus, it is possible that providing a causal explanation (which provides an explanation for why phenomena operate differently than how learners may originally think) may be sufficient enough to induce conceptual change irrespective of the text style (e.g. [50]). Future work will have to disentangle extent to which the text style and the explanation quality could induce conceptual change. We also noted that while the reading level was mostly consistent across all texts, a couple scored as high as the entry level college reading level, a couple others scored as low as the 10^th^ grade reading level. Likewise, a couple texts scored as low as in the 20s for readability while others scored in the 40–50 range. Thus, it is possible that different texts themselves posed challenges to learners. It is also possible that certain concepts were easier to learn than others, and that some individuals were able to learn from the text more easily than others. Future work would have to examine the impact of these factors more closely.

There are a couple of limitations to note in this study. One is that we did not investigate the durability of conceptual change over time. Previous findings have suggested that more conceptual change is evident on delayed posttests after reading refutation text compared to expository (e.g. [34,20,50]). Future work will have to expand on the present findings to see if reading refutation texts results in better retention of newly learned, accurate material compared to expository text. Future work can also investigate whether assessments requiring more elaborate responses (e.g. applying recently learned knowledge as opposed to fact recognition) would show more prominent differences in text style, epistemic beliefs, and conceptual change than multiple-choice questions. Previous work has suggested that transfer task problems as opposed to simple multiple-choice responses seem to provide a better measure of conceptual change after reading text (e.g. [40]); thus, it may be worth investigating how these assessments provide more sensitive measures of students’ conceptual change. Another limitation of this work is that we did not explicitly parcellate the contribution of different models of conceptual change (e.g. knowledge-in-theory versus knowledge-in-pieces). Further testing would be required to examine the contributions of each of these models.

We undertook this work in an effort to expand on previous research suggesting that text type (refutation vs. expository) and epistemic beliefs play a role in driving conceptual change. We did not find evidence that either factor was a strong predictor of conceptual change. At the same time, our exploratory analyses suggest that the nature of academic discipline under study may affect patterns of conceptual change. These findings are based on exploratory analyses and should therefore be treated with caution. Nonetheless, they suggest that the study of conceptual change might involve a more complex set of relationship between different factors than is typically examined in individual research studies.

While the overall amount of conceptual change cannot be readily attributed to text style or epistemic beliefs by themselves, we do observe some finer nuances of those two factors influencing conceptual change on the item and participant levels. Thus, it appears that although individual participants do generally show more conceptual change on posttest performance, the type of text that induces the most conceptual change varies by reader and some items may be challenging to learn than others. Thus, there does not appear to be a single solution to approaching conceptual change; instead, the best method of inducing conceptual change varies for individual student and even across disciplines for those students. These findings suggest that perhaps an individualized approach to instruction and course material would be the best method for inducing conceptual change in science and social science topics.

## Supporting information

S1 Fig Geography task and instructions. The task was identical for the world geography and Floridian geography tasks.
(JPG)

S2 Fig Results for performance test time and text style. (JPG)

S1 File Example text. (PDF)

S2 File Supplemental analyses. Information includes analyses across the five disciplines and includes the figures A-E.
(DOCX)
