# Supplementary figures and images for: Examining the impact of text style and epistemic beliefs on conceptual change
Source: PLoS One. 2019 Sep 4;14(9):e0220766. doi: 10.1371/journal.pone.0220766 (PMC6726218; doi:10.1371/journal.pone.0220766)

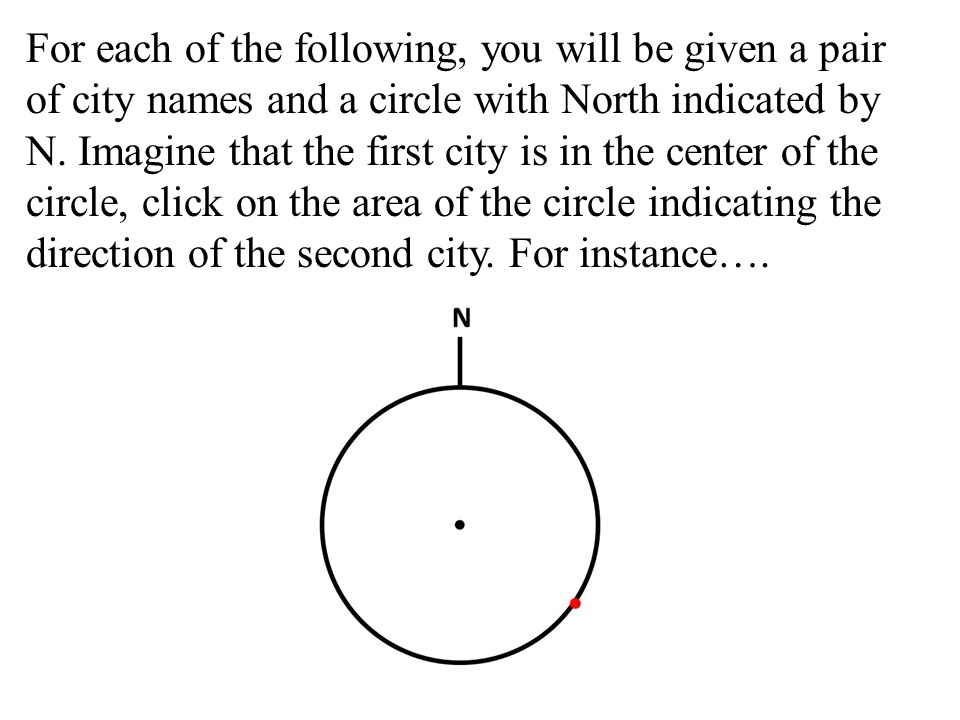

Supplement: S1 Fig — The task was identical for the world geography and Floridian geography tasks. (JPG) [file pone.0220766.s001.jpg]

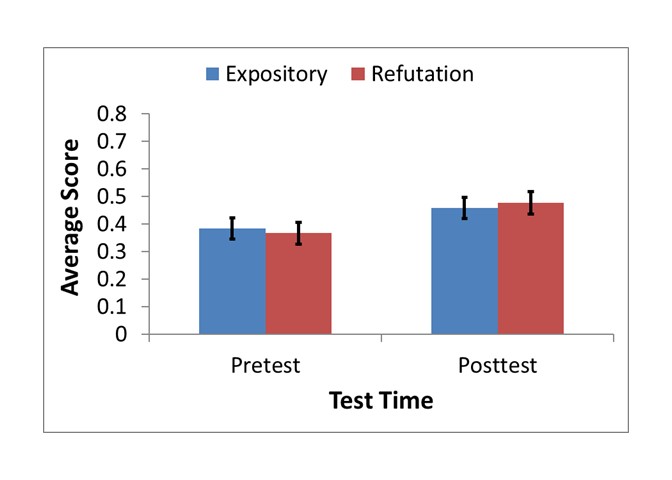

Supplement: S2 Fig — (JPG) [file pone.0220766.s002.jpg]
