# Supplementary material for: Examining the impact of text style and epistemic beliefs on conceptual change
Source: PLoS One. 2019 Sep 4;14(9):e0220766. doi: 10.1371/journal.pone.0220766 (PMC6726218; doi:10.1371/journal.pone.0220766)
Supplement: S1 File — (PDF) [file pone.0220766.s003.pdf]

Introduction Section

When looking at the night sky, one might observe that stars differ in color and brightness. Although stars are widely appreciated for their mysterious beauty, most people do not know what exactly gives stars their color or what those colors signify about the stars themselves. All that we know about stars comes from analyzing the light and radiation that reaches the earth.

Premise Section: Refutation

Many people think red stars are hotter than blue stars because red is often associated with fire and other notably hot surfaces or objects. Astronomers, however, have found this idea to be inaccurate. Quantitative measures of starlight color and stellar temperature reveal that blue stars are actually the hottest stars while red stars are the coolest stars.

Premise Section: Expository

Whether or not blue stars or red stars are the hottest is not that clear to the casual stargazer. Close investigation of the visible light spectrum of stars, however, does reveal vast temperature differences. Astronomers have devised measures for quantifying the colors of light the stars give off and then using those colors to determine stellar temperatures.

Explanation Section

The color of a star provides us with a measure of its surface temperature. In order to determine this, astronomers first have to establish the star's exact color. They do this by measuring the apparent brightness of a star through filters, each of which transmits only the light from a particular narrow band of wavelengths (colors). After they characterize the color of a star, astronomers can determine stellar temperatures using Wien's law, which relates stellar color to stellar temperature. It is now known that very hot stars emit most of their visible light energy at blue wavelengths and cool stars emit their visible light energy as red wavelengths. The hottest

(blue) stars can reach temperatures of around 40,000 K, while the coolest (red) stars have temperatures of about 2,000 K. Another common misconception is that the Sun is the hottest star. Our sun's surface temperature is only about 6,000 K, which explains why it is visibly yellow to us on Earth. Blue and white stars are much hotter than the sun.

Conclusion

While many people do not realize it, the colors of stars have interesting scientific properties, such as the star's temperature. Which color stars are the hottest or the coolest is best determined using quantitative measures developed by astronomers. No two stars are exactly alike in color or temperature, which makes them infinitely fascinating for many nightly stargazers.
