# Supplementary material for: Examining the impact of text style and epistemic beliefs on conceptual change
Source: PLoS One. 2019 Sep 4;14(9):e0220766. doi: 10.1371/journal.pone.0220766 (PMC6726218; doi:10.1371/journal.pone.0220766)
Supplement: S2 File — Information includes analyses across the five disciplines and includes the figures A-E. (DOCX) [file pone.0220766.s004.docx]

**Exploratory analyses by discipline**

While we did not declare that we would examine differences in conceptual change across disciplines in our preregistration, we did nevertheless decide to explore whether the trends observed in the main model are also observed across the individual disciplines of physics, astronomy, genetics, economics, and geography, following the previously described model guidelines for analysis. It is possible that another reason for divergence in whether or not conceptual change was observed in prior work is because different disciplines were examined, such as physics (e.g. 29), evolution (e.g. 47), psychology (e.g. 43), and history (e.g. 21). We also computed scores for texture and variability for each discipline (see Table A for reliabilities).

**Table A. Reliabilities for Texture and Variability for Each Discipline.**

| Discipline | Texture Reliability | Variability Reliability |
| --- | --- | --- |
| Physics | 0.76 | 0.16 |
| Astronomy | 0.70 | -0.07 |
| Genetics | 0.69 | 0.28 |
| Geography | 0.73 | 0.33 |
| Economics | 0.75 | 0.30 |

The main effect of test time found in the main model was also noted in the astronomy (see Table B; Fig. A), physics (see Table C; Fig B), and geography models (see Table D; Fig C), such that participants performed significantly better on the posttest compared to the pretest, which indicates conceptual change within those content areas. Between astronomy, physics, and geography, only astronomy showed an interaction between text style and texture found in the main model. Interestingly, the astronomy model also showed a main effect of variability, although the effect is rather small and should be interpreted with caution. No other main effects or interactions were noted. Physics and geography, on the other hand, showed an interaction between text style and variability that was also observed in the main model. As there are significantly fewer items available for analysis within each discipline model, statistical power is reduced and these findings should be treated as preliminary.

**Table B^a^. Results for Astronomy Model.**

| **Fixed Effects** | | | | | | | | |  |
| --- | --- | --- | --- | --- | --- | --- | --- | --- | --- |
| Predictor | Coefficient | | SE | | t-value | | p-value | |  |
| Intercept | -0.212 | | 0.341 | | -0.622 | | 0.534 | |  |
| Texture | -0.008 | | 0.009 | | -0.847 | | 0.389 | |  |
| **Variability** | **0.033** | | **0.014** | | **2.235** | | **0.020** | |  |
| Text Style | -0.044 | | 0.063 | | -0.696 | | 0.486 | |  |
| **Test Time** | **0.547** | | **0.120** | | **4.546** | | **< 0.001** | |  |
| Text Style x Test Time | 0.066 | | 0.057 | | 1.167 | | 0.243 | |  |
| **Random Effects** | | | | | | | | | |
| Random Effect | | Variance Comp. | | St. Dev. | | χ^2^-value | | p-value | |
| **Text Style x ID** | | **0.163** | | **0.404** | | **270.323** | | **< 0.001** | |
| Test Time x ID | | <0.001 | | 0.018 | | 129.851 | | > 0.500 | |
| Text Time x Test Time x ID | | 0.002 | | 0.047 | | 117.639 | | > 0.500 | |
| Text Style x Item | | 0.017 | | 0.131 | | 11.884 | | 0.219 | |
| **Test Time x Item** | | **0.415** | | **0.173** | | **98.512** | | **< 0.001** | |
| **Text Style x Test Time x Item** | | **0.021** | | **0.143** | | **17.589** | | **0.040** | |
| **Text Style x Texture** | | **<0.001** | | **0.014** | | **16.958** | | **0.049** | |
| Text Style x Variability | | <0.001 | | 0.016 | | 8.302 | | >0.500 | |
| Test Time x Texture | | <0.001 | | 0.007 | | 9.413 | | 0.400 | |
| Test Time x Variability | | <0.001 | | 0.012 | | 4.990 | | >0.500 | |
| Text Style x Test Time x Texture | | <0.001 | | 0.012 | | 9.874 | | 0.361 | |
| Text Style x Test Time x Variability | | <0.001 | | 0.015 | | 4.112 | | > 0.500 | |

^a^ID refers to participant.

**
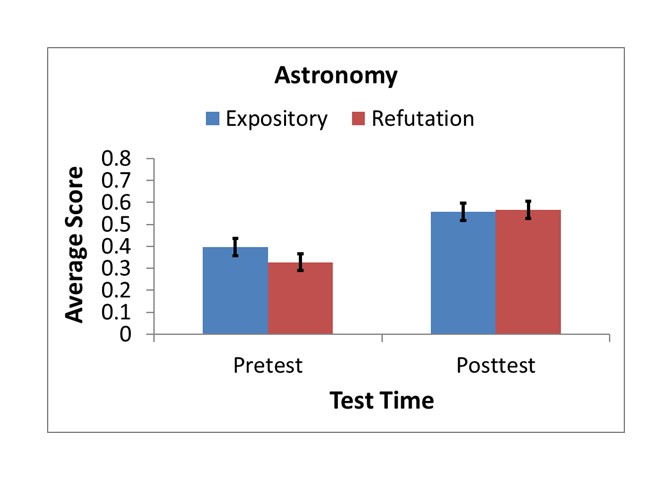
**

**Fig A. Results for Performance Test Time and Text Style in Astronomy Model.**

**Table C^a^. Results for Physics Model.**

| **Fixed Effects** | | | | | | | |
| --- | --- | --- | --- | --- | --- | --- | --- |
| Predictor | Coefficient | | SE | | | t-value | p-value |
| Intercept | -0.919 | | 0.244 | | | -3.772 | <0.001 |
| Texture | 0.001 | | 0.006 | | | 0.216 | 0.829 |
| Variability | 0.002 | | 0.011 | | | 0.200 | 0.842 |
| Text Style | -0.021 | | 0.064 | | | -0.325 | 0.745 |
| **Test Time** | **0.282** | | **0.135** | | | **2.088** | **0.037** |
| Text Style x Test Time | 0.068 | | 0.064 | | | 1.054 | 0.292 |
| **Random Effects** | | | | | | | |
| Random Effect | | Variance Comp. | | St. Dev. | χ^2^-value | | p-value |
| **Text Style x ID** | | **0.192** | | **0.438** | **309.998** | | **< 0.001** |
| Test Time x ID | | 0.006 | | 0.0778 | 113.576 | | >0.500 |
| Text Style x Test Time x ID | | 0.002 | | 0.046 | 93.742 | | >0.500 |
| **Text Style x Item** | | **0.016** | | **0.127** | **22.643** | | **0.012** |
| **Test Time x Item** | | **0.391** | | **0.153** | **65.992** | | **< 0.001** |
| Text Style x Test Time x Item | | 0.124 | | 0.015 | 12.145 | | 0.144 |
| Text Style x Texture | | <0.001 | | 0.010 | 15.502 | | 0.114 |
| **Text Style x Variability** | | **<0.001** | | **0.027** | **25.581** | | **0.005** |
| Test Time x Texture | | <0.001 | | 0.006 | 5.921 | | > 0.500 |
| Test Time x Variability | | <0.001 | | 0.013 | 4.985 | | >0.500 |
| **Text Style x Test Time x ID** | | **0.035** | | **0.187** | **35.223** | | **<0.001** |
| Text Style x Test Time x Texture | | <0.001 | | 0.004 | 3.152 | | >0.500 |
| Text Style x Test Time x Variability | |  | | 0.016 | 11.897 | | 0.291 |

**
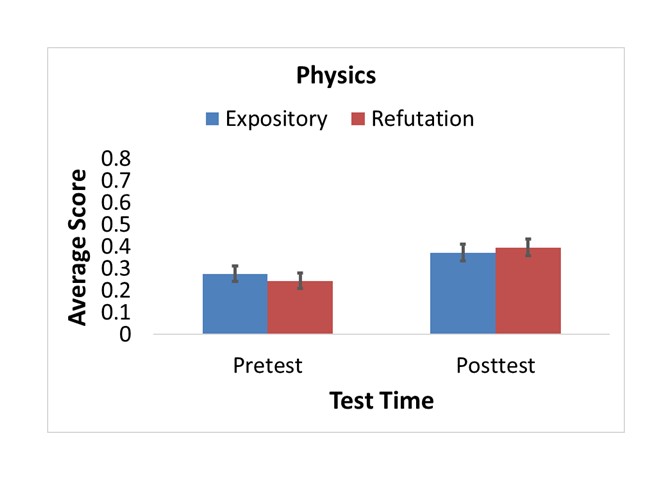
**

**Fig B. Results for Performance Test Time and Text Style in Physics Model.**

**Table D^a^. Results for Geography Model.**

| **Fixed Effects** | | | | | | | |  |  |
| --- | --- | --- | --- | --- | --- | --- | --- | --- | --- |
| Predictor | Coefficient | SE | | | t-value | | p-value |  |  |
| Intercept | -0.359 | 0.170 | | | -2.120 | | 0.034 |  |  |
| Texture | -0.003 | 0.007 | | | -0.409 | | 0.683 |  |  |
| Variability | -0.010 | 0.009 | | | -1.134 | | 0.259 |  |  |
| Text Style | -0.004 | 0.056 | | | -0.064 | | 0.949 |  |  |
| **Test Time** | **0.161** | **0.072** | | | **2.232** | | **0.026** |  |  |
| **Text Style x Test Time** | **0.072** | **0.031** | | | **2.335** | | **0.020** |  |  |
| **Random Effects** | | | | | | | | | |
| Random Effect | | Variance Comp. | | St. Dev. | χ^2^-value | | p-value | | |
| **Text Style x ID** | | **0.175** | | **0.418** | **367.597** | | **<0.001** | | |
| Test Time x ID | | 0.009 | | 0.093 | 131.061 | | >0.500 | | |
| Text Style x Test Time x ID | | 0.005 | | 0.072 | 119.538 | | >0.500 | | |
| **Text Style x Item** | | **0.020** | | **0.143** | **33.442** | | **0.004** | | |
| **Test Time x Item** | | **0.078** | | **0.279** | **105.750** | | **<0.001** | | |
| Text Style x Test Time x Item | | 0.001 | | 0.038 | 5.394 | | >0.500 | | |
| Text Style x Texture | | <0.001 | | 0.013 | 21.316 | | 0.127 | | |
| **Text Style x Variability** | | **<0.001** | | **0.030** | **53.332** | | **<0.001** | | |
| Test Time x Texture | | <0.001 | | 0.002 | 5.620 | | >0.500 | | |
| Test Time x Variability | | <0.001 | | 0.005 | 9.541 | | >0.500 | | |
| Text Style x Test Time x Texture | | <0.001 | | 0.008 | 14.021 | | >0.500 | | |
| Text Style x Test Time x Variability | | <0.001 | | 0.008 | 6.350 | | >0.500 | | |

^a^ID refers to participant.


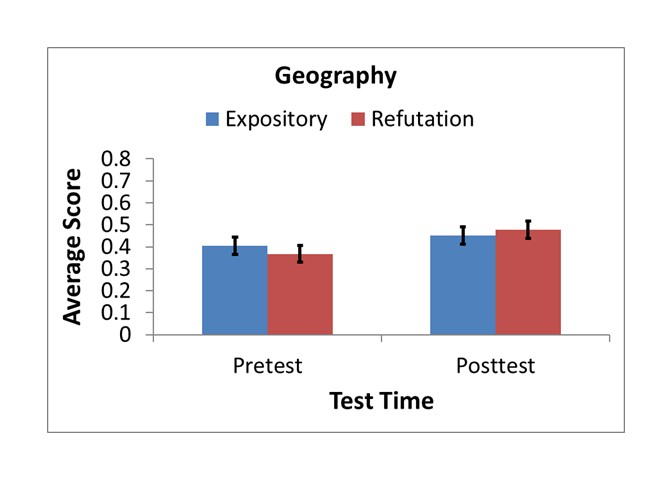


**Fig C. Results for Performance Test Time and Text Style in Geography Model.**

Contrary to what we found in the main model, there was no main effect of test time in the genetics model (see Table E; Fig D) and economics models (see Table F; Fig E). Both models, However, the genetics model did show an interaction between text style and variability, that was also present in the main model. Diverging from the main model, both the genetics and economics models showed a main effect of variability, such that the higher a learner scored in variability, the more conceptual change they exhibited. Again, these effects are rather small and should be interpreted with caution. The economics model also showed a two-way interaction between text style and test time, and a three-way interaction between text style, test time, and variability. Because this interaction is rather small, however, these results should be interpreted with caution and treated as preliminary.

**Table E^a^. Results for Genetics Model.**

| **Fixed Effects** | | | | |
| --- | --- | --- | --- | --- |
| Predictor | Coefficient | SE | t-value | p-value |
| Intercept | -1.080 | 0.313 | -3.453 | <0.001 |
| Texture | -0.007 | 0.010 | -0.690 | 0.491 |
| **Variability** | **0.022** | **0.013** | **1.722** | **0.087** |
| Text Style | 0.068 | 0.087 | 0.775 | 0.439 |
| Test Time | 0.021 | 0.125 | 1.168 | 0.867 |
| Text Style x Test Time | 0.014 | 0.053 | 0.269 | 0.788 |
| **Random Effects** | | | | |
| Random Effect | Variance Comp. | St. Dev. | χ^2^-value | p-value |
| **Text Style x ID** | **0.055** | **0.235** | **35.283** | **< 0.001** |
| Test Time x ID | 0.001 | 0.033 | 82.315 | >0.500 |
| Text Style x Test Time x ID | 0.001 | 0.032 | 67.393 | >0.500 |
| Text Style x Item | 0.124 | 0.015 | 13.400 | 0.098 |
| **Test Time x Item** | **0.391** | **0.153** | **65.993** | **< 0.001** |
| Text Style x Test Time x Item | 0.124 | 0.015 | 12.145 | 0.144 |
| Text Style x Texture | <0.001 | 0.015 | 7.142 | > 0.500 |
| **Text Style x Variability** | **<0.001** | **0.019** | **16.797** | **0.032** |
| Test Time x Texture | <0.001 | 0.010 | 7.142 | > 0.500 |
| Test Time x Variability | <0.001 | 0.016 | 7.590 | >0.500 |
| **Text Style x Test Time X ID** | **0.012** | **0.107** | **16.136** | **0.040** |
| Text Style x Test Time x Texture | <0.001 | 0.003 | 2.434 | >0.500 |
| Text Style x Test Time x Variability | <0.001 | 0.005 | 5.208 | > 0.500 |

^a^ID refers to participant.

**
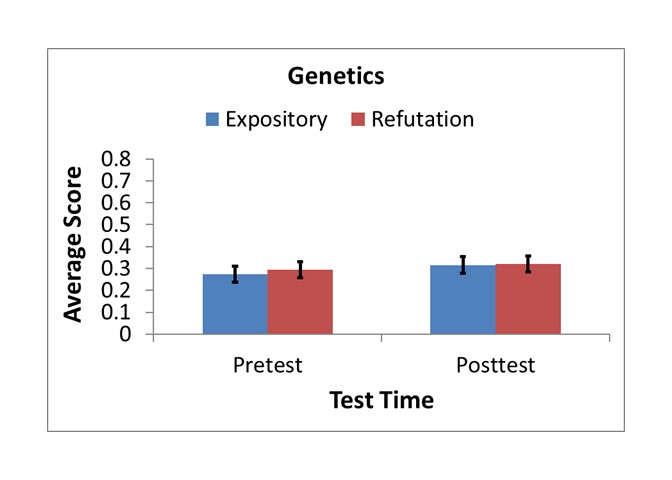
**

**Fig D. Results for Performance Test Time and Text Style Genetics Model.**

**Table F^a^. Results for Economics Model.**

| **Fixed Effects** | | | | | | | | |
| --- | --- | --- | --- | --- | --- | --- | --- | --- |
| Predictor | | Coefficient | SE | | t-value | | p-value | |
| Intercept | | 0.886 | 0.461 | | 1.923 | | 0.055 | |
| Texture | | -0.015 | 0.014 | | -1.095 | | 0.275 | |
| **Variability** | | **0.048** | **0.024** | | **2.001** | | **0.047** | |
| Text Style | | 0.143 | 0.116 | | 1.231 | | 0.219 | |
| Test Time | | 0.173 | 0.147 | | 1.178 | | 0.239 | |
| **Text Style x Test Time** | | **-0.187** | **0.082** | | **-2.279** | | **0.023** | |
| **Random Effects** | | | | | | | | |
| Random Effect | | Variance Comp. | | St. Dev. | χ^2^-value | | p-value | |
| **Text Style x ID** | | **1.099** | | **1.201** | **659.847** | | **<0.001** | |
| Test Time x ID | | 0.377 | | 0.124 | 147.087 | | >0.500 | |
| **Text Style x Test Time x ID** | | **0.486** | | **0.236** | **191.966** | | **0.018** | |
| **Text Style x Item** | | **0.163** | | **0.027** | **15.748** | | **0.027** | |
| **Test Time x Item** | | **0.419** | | **0.176** | **66.650** | | **<0.001** | |
| **Text Style x Test Time x Item** | | **0.153** | | **0.023** | **12.802** | | **0.076** | |
| Text Style x Texture | | 0.012 | | <0.001 | 4.814 | | >0.500 | |
| Text Style x Variability | | 0.028 | | <0.001 | 8.135 | | 0.320 | |
| Test Time x Texture | | 0.017 | | <0.001 | 10.589 | | 0.157 | |
| Test Time x Variability | | 0.021 | | <0.001 | 6.606 | | >0.500 | |
| **Text Style x Test Time x Texture** | | **0.024** | | **<0.001** | **14.591** | | **0.041** | |
| Text Style x Test Time x Variability | | 0.032 | | 0.001 | 11.875 | | 0.104 | |

^a^ID refers to participant.

**
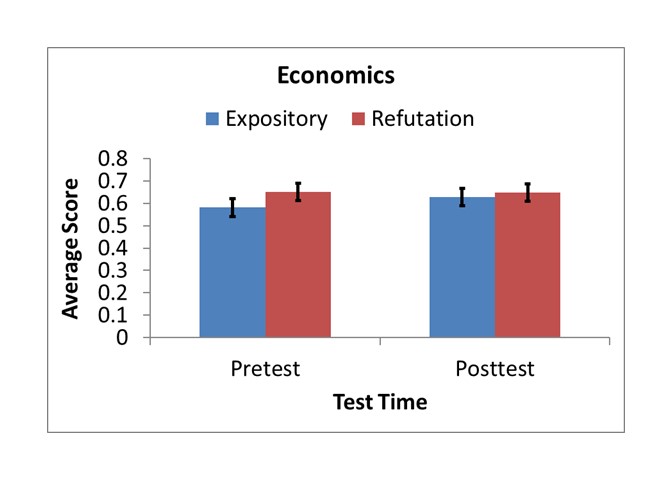
**

**Fig E. Results for Performance Test Time and Text Style Economics Model.**

Overall, the effects are not uniform across the five disciplines. These differences could be attributed to a number of factors. On the item level, certain items, topics, or disciplines may have been easier or more difficult for learners. On the participant level, readers may learn better within the context of the experiment. There were also significantly fewer items within each discipline model (ranging from 10-18 items) compared to the main model (containing 64 items), thus reducing the power and measurement precision. Nevertheless, these exploratory analyses reveal that differences across disciplines may also impact the extent to which conceptual change is observed, and which factors drive this process. Despite these differences, a couple similarities should be noted across disciplines. One is that for astronomy, physics, and geography, there was a main effect of test time such that participants performed significantly better on the posttest compared to the pretest. Two is that geography and economics both shared an interaction of text style and test time. There are weak interactions between text style and variability for physics, geography, and genetics suggesting interplay between text style and an aspect of epistemic beliefs for those disciplines, but future studies would have to see if these interactions could be replicated.
